# Comparative studies on multi-carrier transmission schemes in mountainous and dense forest environment

**DOI:** 10.1038/s41598-022-20895-0

**Published:** 2022-10-07

**Authors:** Chuanfei Ding, Xiang Gao, Kai Yang, Jinpeng Song, Ruide Li, Xiangyuan Bu, Jianping An

**Affiliations:** 1 School of Information and Electronics, Beijing Institute of Technology, Beijing, 100081 China; 2 School of Cyberspace Science and Technology, Beijing Institute of Technology, Beijing, 100081 China

**Keywords:** Electrical and electronic engineering, Natural hazards

## Abstract

Earthquakes, forest fires, mudslides and other natural disasters occur frequently in recent years. They usually occur in the mountainous and dense forests, where local communication facilities do not exist or have been destroyed by the disasters. Adverse geographical environment poses a huge challenge to emergency communications and rescue. This paper presents comparative studies on multi-carrier transmission schemes in the mountainous and dense forest environment. The comprehensive communication performance for various multi-carrier waveform schemes, has been extensively analyzed by using the Stanford University Interim channel model. Simulation results show that the pruned discrete Fourier transform spread filter bank multi-carrier scheme exhibits generally the best performance in terms of transmission rate and distance for most operation modes.

## Introduction

Natural disasters such as the forest fires, earthquakes and mudslides, pose significant threats to human health and safety year by year. For the mountainous and dense forests, forest fires are the most frequent hazards due to the special weather and vegetation conditions. For example, a large forest fire was caused by the lightning strike in Muli County of Sichuan Province, China in 2019. The conflagration zone is a mountainous area filled with deep valleys and dense trees at an altitude of about 3800 m. Poor geographical conditions hinder the construction of local communication facilities, which make the firefighters out of contact with each other thus causing huge casualties. Therefore, a robust emergency communication system is of critical importance to the mountainous and dense forest environment, which allows the front-line rescuers to timely know where there is a danger and how to protect the lives.

Emergency communication relevant technologies have been investigated in the past decades. Various methods were proposed for disaster management and early warning. Based on those wireless sensor network models^1–3^, the forest fires can be effectively managed and monitored to a certain extent. In response to the shortage of communication resources of at the disaster sites, big data-based analytics are utilized to enhance the robustness of emergency communication networks^4^. Those reported studies which focus on disaster perception and post-disaster relief management are mainly network-layer technologies for emergency rescue in the forest environment. On the other hand, some researchers have investigated the electromagnetic wave propagation characteristics in the forests. It was found that the spatial diversity of radio waves from the receiver location helps to improve the average gain^5^. A simulation platform^6^ was developed for identifying the radio exclusion zones due to the cold plasma caused by the forest fires. Currently reported studies mainly focus on either resource management or electromagnetic propagation in emergency communication scenarios, which never discuss critical underlying communication issues like communication scheme, waveform design, channel model and link performance (e.g., the bit error ratio (BER), data rate and transmission distance).

In the mountainous and dense forests, the presence of various obstacles and high-density vegetation lead to severe propagation losses, and the complex topography bring multipath effects to the wireless links. Faced with severe multipath fading, conventional single-carrier transmission systems struggle to achieve high speed transmission. To solve the multipath problem, we propose in this work to apply the multi-carrier transmission scheme to emergency communication in the mountainous and dense forests. At an earlier time, Weinstein and Ebert developed the orthogonal frequency division multiplexing (OFDM) system^7^ using the discrete Fourier transform (DFT) technique to simplify computation. As a multi-carrier transmission scheme, the OFDM scheme has widely been investigated due to its ease of implementation, superior anti-multipath performance and relatively high spectral efficiency^8–10^. However, OFDM systems generally suffer from relatively high peak-to-average power ratio (PAPR)^11,12^, which inevitably degrades the transmitting power efficiency^13,14^ and thus deteriorates the signal-to-noise ratio (SNR) at the receivers, especially for emergency communication applications. To reduce the PAPR of OFDM systems, Long Term Evolution (LTE) developed the DFT spread OFDM (DFT-s-OFDM) technique^15^, which exhibits the single-carrier PAPR characteristics while preserving the advantages of OFDM. In response to the out-of-band (OOB) emissions problem of DFT-s-OFDM or Cyclic Prefix-OFDM (CP-OFDM), Berardinelli and Tavares proposed the Zero-Tail DFT spread OFDM (ZT-DFT-s-OFDM) scheme^16,17^. By adding a certain length of zero-head (ZH) at the beginning and zero-tail (ZT) at the end of each symbol, respectively, one smoothens the in-band and out-band transitions thus reducing the OOB signal emissions.

In recent years, the filter bank multi-carrier offset quadrature amplitude modulation (FBMC-OQAM)^18^ communication technology has attracted much attention due to its more flexible time–frequency resource allocation and excellent OOB emissions suppression capability as compared with those OFDM based communication schemes^19,20^. In the OFDM system, a DFT spreading is added before the modulation of the inverse fast Fourier transform (IFFT) to reduce the PAPR. Such an idea was then rightfully applied to the FBMC system as well, but it was found that due to the special characteristics of FBMC-OQAM system, the simple DFT spread FBMC-OQAM (hereafter called Simple DFT-s-FBMC) cannot fully restore its PAPR to a satisfactory level^21^. Therefore, a number of researchers have made some improvements to the Simple DFT-s-FBMC scheme, e.g., proposing a selection scheme based on the influence of phase term^22^. However, this scheme requires additional side-information (SI) which increases the complexity and reduces the transmission efficiency. Ronald Nissel proposed a pruned DFT spread FBMC-OQAM schemes (hereafter called Pruned-DFT-s-FBMC)^23^, which combines the advantages of both low PAPR characteristics and high time–frequency localization (TFL).

Despite that abundant theoretical analyses have been conducted on those multi-carrier schemes for general case, there is still no study on the multi-carrier transmission scheme in combination with special channel conditions or scenario setups in the mountainous and dense forest environment. There is no discussion about specific communication solutions or channel models used for different emergency scenarios, not even to mention what multi-carrier scheme is best suited to what scenario in such specific environment. In this work, our innovative contributions are highlighted below. This paper presents extensive comparative studies on various multi-carrier communication schemes when applied in the mountainous and dense forest scenarios. More specifically, two communication solutions are proposed for emergency rescue in the mountainous and dense forest. One approach is the point-to-point communication between the ground rescue personals carrying the equipment, which is a relatively simple link with short transmission distance. Another way is the auxiliary communication based on the aerial base stations, which features more complex links while gaining more benefits. For those two communication solutions, we propose the studies of the Stanford University Interim (SUI) channel model in combination with different multi-carrier schemes for mountainous and dense forest communications. In particular, the comprehensive communication performance is extensively analyzed and comparatively studied for various multi-carrier waveform schemes. By setting the wireless system parameters as similar as that in practical engineering applications, the transmission distance and rates are analyzed in detail with the power spectrum density (PSD), PAPR and BER for various multi-carrier waveforms used in different operation modes, which shows that the Pruned-DFT-s-FBMC scheme exhibits generally the best performance for most modes.

### Solutions and models

**Figure 1:**
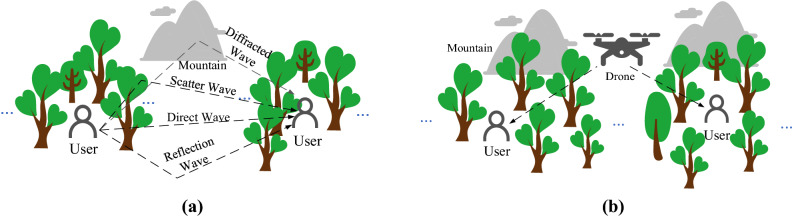
Two communication solutions for the mountainous and dense forest environment: (**a**) the point-to-point link between the rescuers carrying rescue equipment to communicate with each other; (**b**) the UAV air-assisted communication link.

Two communication solutions are proposed for emergency rescue applications in the mountainous and dense-forest environment. As depicted in Fig. 1a, we consider a number of rescuers carrying backpacking rescue equipment in the dense forest to perform point-to-point communication between each other. The point-to-point wireless networks generally feature simple link construction, flexible networking and relatively low system cost in engineering applications. However, complex multipath environment and severe path losses affect the signal transmission seriously. As shown in Fig. 1a, the propagation paths in the mountainous and dense forest generally include the direct waves, the reflected waves from the ground, the scattered waves from the trees and the diffracted waves from the mountains. Moreover, due to the restriction of antenna volume for personnel carry equipment, the signal transmission distance is significantly limited. To solve this problem, we propose an alternative solution called unmanned aerial vehicle (UAV) air-assisted communication as depicted in Fig. 1b. Advanced conformal antennas of large size and high gain can be developed on those UAVs for increasing the communication range. Besides, wide range search and fast rescue can be achieved via beam forming techniques. Nevertheless, the fast-moving UAVs cause relatively high Doppler shift imposing high challenge on communication performance. For the second solution, we consider both the high-speed and low-speed motion modes of the UAVs as well as whether to use the antennas with directional beams.

Considering the wireless propagation characteristics in the mountainous and dense-forest environment, the SUI channel model^24^ is applied for the first time to investigating multi-carrier waveform schemes for such scenario. According to the tree density and path loss amount, the terrain is classified into three categories A, B and C in the SUI channel model, corresponding to that the hilly terrain with moderate to heavy tree density, the hilly terrain with light tree density or the flat terrain with moderate to heavy tree density, and the mostly flat terrain with light tree density, respectively. Taking into consideration the magnitude of the time delay extension and Doppler frequency shift, there are a total of six SUI channel sub-models. Considering the high time delay characteristics, we choose terrain A together with the SUI-5 and SUI-6 sub-modes to simulate the mountainous and dense forest environment. The difference between those two sub-modes is whether the Doppler is low or high. Thus, the SUI-5 channel is corresponding to the first solution and the second solution where the UAV is in low-speed movement, and the SUI-6 channel is corresponding to the second solution for high-speed moving UAV.

There are many factors that affects the path loss, such as the height and gain of the transceiver antennas, carrier frequency and geographical environment. The path loss for the SUI model is calculated as^25^

1 $$PL(d)_{dB} = \left\{ \begin{array}{*{20}l} {\text{20log}}_{10} \left( {\frac{{4\pi d_{0}^{^{\prime}} c}}{{Gf_{c} }}} \right) + 10\gamma \log_{10} \left( {\frac{d}{{d_{0} }}} \right) + C_{f} + C_{r} ,d > d_{0} \hfill \\ {\text{20log}}_{10} \left( {\frac{4\pi dc}{{Gf_{c} }}} \right),d \le d_{0} \hfill \\ \end{array} \right.,$$

where *c, f*_*c*_*, G* and *d* are the light speed, carrier frequency, antenna gain and transmission distance, respectively. The parameters $\gamma$, *C*_*f*_*, C*_*r*_ and $d_{0}^{^{\prime}}$ in (1) are the correction factors for the terrain, carrier frequency, antenna height and reference distance at the receiver, respectively, which are denoted as

2 $$\gamma = a - bh_{tx} + c/h_{tx} ,$$

3 $$C_{f} = 6\log_{10} (f_{c} /2000),$$

4 $$C_{r} = - 10.8\log_{10} (h_{rx} /2),$$

5 $$d_{0}^{^{\prime}} = d_{0} 10^{{ - (C_{f} + C_{r} )/10\gamma }} .$$

In (2) and (4), *h*_*tx*_ and *h*_*rx*_ are the antenna height of the transmitter and receiver, respectively; *a*, *b* and *c* are determined by the terrain, which have the values of 4.6, 0.0075 and 12.6, respectively. *d*_*0*_ is a variable in the reference distance and takes the value of 100 m.

**Figure 2:**
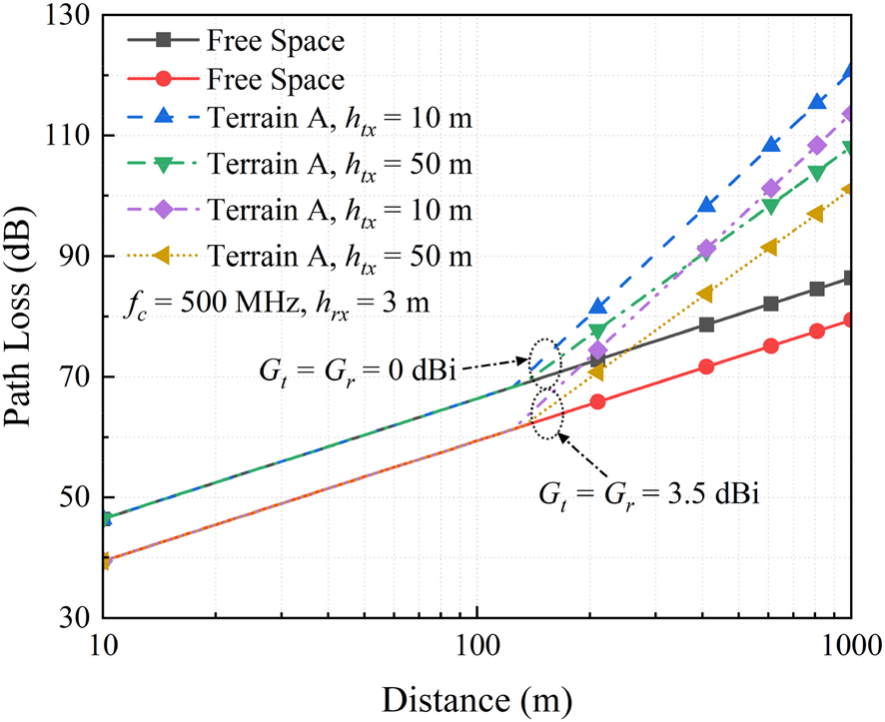
Simulated path loss versus communication distance in the mountainous and dense forest environment.

Figure 2 shows the simulated path loss versus communication distance for a wireless link in the mountainous and dense forest environment. Here, the carrier frequency *f*_*c*_ and receiving antenna height *h*_*rx*_ are kept constants, while the transmitting antenna height *h*_*tx*_ and the gain of both antennas (i.e., *G*_*t*_ and *G*_*r*_) are changed. It can be clearly seen that, the path loss of terrain A is almost as same as that in the free space for a relatively shorter range of about 120 m, which is the reference distance. In other words, the propagation link over a distance less than the reference distance can be approximated to be a free space link. As the propagation distance exceeds the reference distance, a sharp increase is observed for the path loss curves other than free space curves. In fact, this dramatic increase in path loss is due to the dense vegetation and relatively high air humidity in the mountainous and forests. On the other hand, the transmitting antenna height plays a significant role on the slope of those path curves, where the path loss for a 50 m-high antenna is about 15 dB lower than that for a 10 m-high one. Furthermore, higher antenna gain is always beneficial to reducing the path loss. Generally, it is impractical for the rescuers to carry 50 m or higher antennas. Hence, we propose a second solution that UAV-assisted communication, which takes advantage of the UAV height thus raising the height of the transmitting antenna conformal to the UAV bodies. In this situation, when the transmission distance is one kilometer, the link path loss for the mountainous and dense forest environment is about 120 dB, which is 40 dB higher than free space.

A well-performing prototype filter determines the overall performance of the signal. For this purpose, several filters commonly used in the OFDM and FBMC based schemes are studied by analyzing their TFL as described by an ambiguity function^19^

6 $$A(\tau ,v) = \int_{ - \infty }^{\infty } {p_{tx} (t - \pi /2)} p^{*}_{rx} (t + \tau /2)e^{j2\pi vt} dt,$$

where *p*_*tx*_*(t)* and *p*_*rx*_*(t)* represent the shaping and matched filters, respectively. According to the matched filter theory, those two filters usually take the same form. The variables *τ* and *v* are the time and frequency offsets, respectively. As a two-dimensional correlation function of the time and frequency, the ambiguity function *A*(*τ*, *v*) measures the ability of the signal against inter-symbol interferences (ISIs) and inter-carrier interferences (ICIs)^26^. The OFDM systems that employ the rectangular filters generally feature relatively low computational complexities. However, such simple rectangular prototype filters suffer from poor localization in the frequency domain. This explains why OOB emission for the OFDM based systems is extremely serious. In around 2010, another prototype filter called PHYDYAS^27^ was proposed by an EU research institute, which has the expression of^22^

7 $$p(t) = \left\{ {\begin{array}{*{20}l} {1 + 2\sum\limits_{i = 1}^{O - 1} {b_{i} \cos \left( {\frac{2\pi t}{{OT_{0} }}} \right)} } & { - \frac{{OT_{0} }}{2} < t < \frac{{OT_{0} }}{2}} \\ 0 & {otherwise} \\ \end{array} } \right.,$$

where the coefficients *b*_*i*_ and *T*_*0*_ are determined by the overlapping factor *O* (generally with the value of 4 or 8)^27^ and desired subcarrier spacing, respectively. Setting the factor *O* to be 4, the coefficient *b*_*i*_ would be

8 $$b_{1} = 0.97195983;b_{2} = \sqrt 2 /2;b_{3} = 0.23514695.$$

Another popular filter is called Hermite prototype filter, which is constructed by^28^

9 $$p(t) = \frac{1}{{T_{0} }}e^{{ - 2\pi \left( {\frac{t}{{T_{0} }}} \right)^{2} }} \sum\nolimits_{{i = \{ 0,4,8,12,16,20\} }} {a_{i} } H_{i} \left( {2\sqrt \pi \frac{t}{{T_{0} }}} \right),$$

where the coefficient ${a}_{i}$ is found to be^29^

10 $$\begin{gathered} a_{0} = 1.412692577,\;a_{4} = - 3.0145 \times 10^{ - 3} ,\;a_{8} = - 8.8041 \times 10^{ - 6} \hfill \\ a_{12} = - 2.2611 \times 10^{ - 9} ,\;a_{16} = - 4.457 \times 10^{ - 15} ,\;a_{20} = 1.8633 \times 10^{ - 16} . \hfill \\ \end{gathered}$$

In (9), *H*_i_ (·) denotes the Hermite polynomials that are derived from the solution of the Hermite equation, i.e.,

11 $$y^{\prime\prime} - 2xy^{\prime} + 2ny = 0.$$

The solution to (11) is generally in the differential form as

12 $$H_{n} (x) = ( - 1)^{n} e^{{x^{2} }} \frac{{d^{n} e^{{ - x^{2} }} }}{{dx^{n} }},$$

which requires further derivation calculations to arrive at the final desired intuitive form of the expression. To improve the calculation efficiency for higher-order filters, the solution of the Hermite equation is derived in the form of series

13 $$H_{n} (x) = \sum\limits_{m = 0}^{[n/2]} {( - 1)^{n} \frac{n!}{{m!(n - 2m)!}}(2x)^{n - 2m} } ,$$

where [·] denotes the operation of rounding.

**Figure 3:**
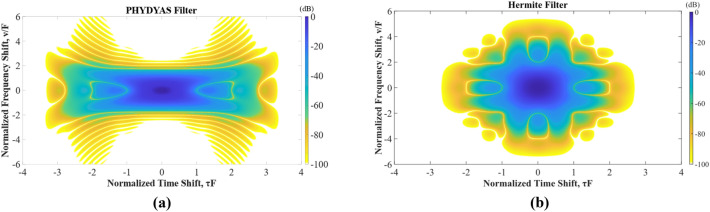
Simulated ambiguity functions of the (**a**) PHYDYAS filter and (**b**) Hermite filter.

Figure 3 shows the simulated ambiguity function of the PHYDYAS and Hermite prototype filters. As depicted in Fig. 3a, the dark color is more concentrated on the vertical axis and relatively scattered on the horizontal axis, which shows that the PHYDYAS filter exhibits excellent frequency-domain localization but relatively poor time localization. In comparison, the Hermite filter exhibits a more compromised TFL characteristics as illustrated in Fig. 3b. Clearly, the designed 20th order Hermite filter has better time localization, but slightly worse frequency localization than that of the PHYDYAS filter. Considering the complicated channel conditions in the mountainous and dense-forest environment, the Hermite filter has a more appropriate TFL performance and thus would be more robust for communication applications.

## Results

### PAPR and OOB emission analyses

**Figure 4:**
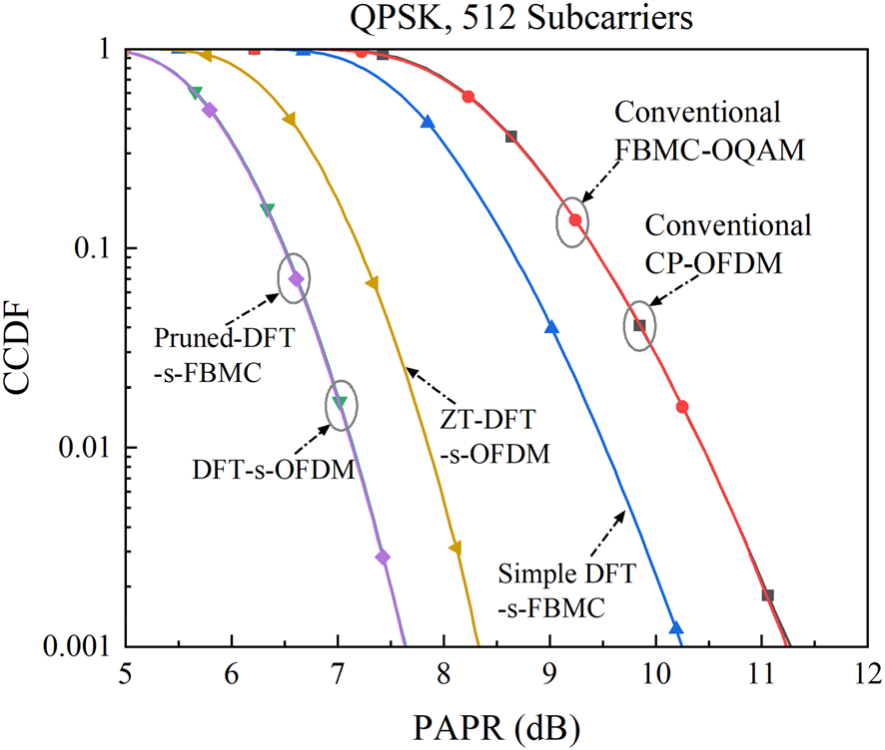
Simulated PAPR of different multi-carrier signal waveforms.

Figure 4 shows the simulated PAPR of different multi-carrier signal waveforms. The complementary cumulative distribution function (CCDF) is used to quantify the PAPR in a multi-carrier transmission system that exceeds a certain threshold value^30^, typically 10^–3^. The modulation mode of quadrature phase shift keying (QPSK) is used for a fair comparison. It is seen that the conventional CP-OFDM and FBMC-OQAM schemes exhibit the same level of extremely poor PAPR performance. Due to the effect of the OQAM phase shift factor, simple DFT spreading imposed on the FBMC-OQAM scheme can only reduce the PAPR by around 1 dB. The ZT-DFT-s-OFDM scheme features a special symbol design that each symbol starts and ends with a low power ZT or ZH connection, for which the PAPR is 3 dB lower than that of the conventional CP-OFDM. In comparison, the DFT-s-OFDM and Pruned-DFT-s-FBMC schemes exhibit the lowest PAPR, which is 0.6 dB lower than that of the ZT-DFT-s-OFDM scheme due to their relatively higher signal average power.

**Figure 5:**
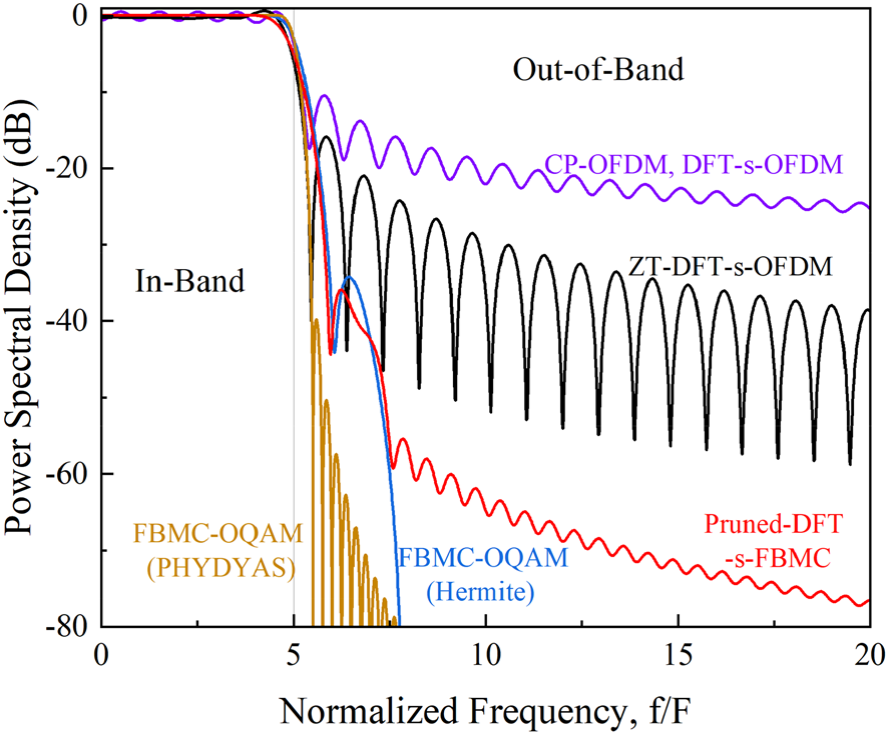
Simulated PSD of different multi-carrier signal waveforms.

Figure 5 shows the simulated PSD of different multi-carrier signal waveforms. Clearly, all multi-carrier schemes exhibit relatively flat in-band response but different OOB emission levels. Due to the use of rectangular window with extremely poor frequency localization, there exists most severe OOB emissions for the conventional CP-OFDM and DFT-s-OFDM signals. Using a similar rectangular window, the OOB emission of the ZT-DFT-s-OFDM system can be reduced by about 12 dB as compared with those conventional ones, which mainly benefits from the relatively smooth in-band and OOB transitions with the addition of ZH and ZT before and after each symbol. For the FBMC-OQAM schemes, using a PHYDYAS filter is the most effective way to achieve low OOB emission, which can be around 60 dB lower than that of the CP-OFDM scheme. This is due to the excellent frequency localization of the PHYDYAS filter. Finally, it is noticed that although the OOB emission of the Pruned-DFT-s-FBMC signal is slightly higher, but still 25 dB lower than that of the CP-OFDM and DFT-s-OFDM schemes. In fact, such level of OOB emission is sufficient for most practical applications.

### Bit error ratio (BER) performance analysis

The BER performance has been investigated for multi-carrier communication schemes in the mountainous and dense forest environment. Simple one-tap minimum mean square error (MMSE) frequency domain equalization^31,32^ are performed for all those multi-carrier schemes. For the first communication solution for emergency rescue, the SUI-5 channel model is used for link simulation and analysis. Considering the communication links are built between walking rescuers in the mountainous and dense forest environment, it is assumed that the rescuers are equipped with omnidirectional antennas with limited volume. Besides, assuming that the rescuers move at a speed of 1 m/s (equal to 3.6 km/h), the resultant Doppler shift effect is little and thus can be ignored.

The power delay profile (PDP) is commonly used to describe the multipath fading channels^33^. More specifically, the PDP includes the parameters of the time delay and power over different channels. Table 1 shows the PDPs of the SUI-5 and SUI-6 channel models for the cases of using the omnidirectional and 30° directional antennas, respectively. Due to the severe multipath effect in the mountainous and dense forest scenarios, the SUI-5 channel has a maximum multipath delay of 10 µs, which is longer than most of the 3GPP’s delay channel. Assuming that the subcarrier spacing *F* is 15 kHz (typical value for the multi-carrier systems), the time length of each OFDM or FBMC symbol is thus *T* = 1/*F* = 66.6 µs. The time lengths of the CP and ZT (including ZH) are both set to be *T*/4, which are long enough for against the multipath effect but at the sacrifice of spectrum efficiency.

**Table 1:** The PDPs of the SUI-5 and SUI-6 channels.

Channel	Parameters	Tap 1	Tap 2	Tap 3
SUI-5	Time delay (µs)	0	4	10
	Channel power (Omni. antenna) (dB)	0	− 5	− 10
	Channel power (30°antenna) (dB)	0	− 11	− 22
SUI-6	Time delay (µs)	0	14	20
	Channel power (Omni. antenna) (dB)	0	− 10	− 14
	Channel power (30° antenna) (dB)	0	− 16	− 26

**Figure 6:**
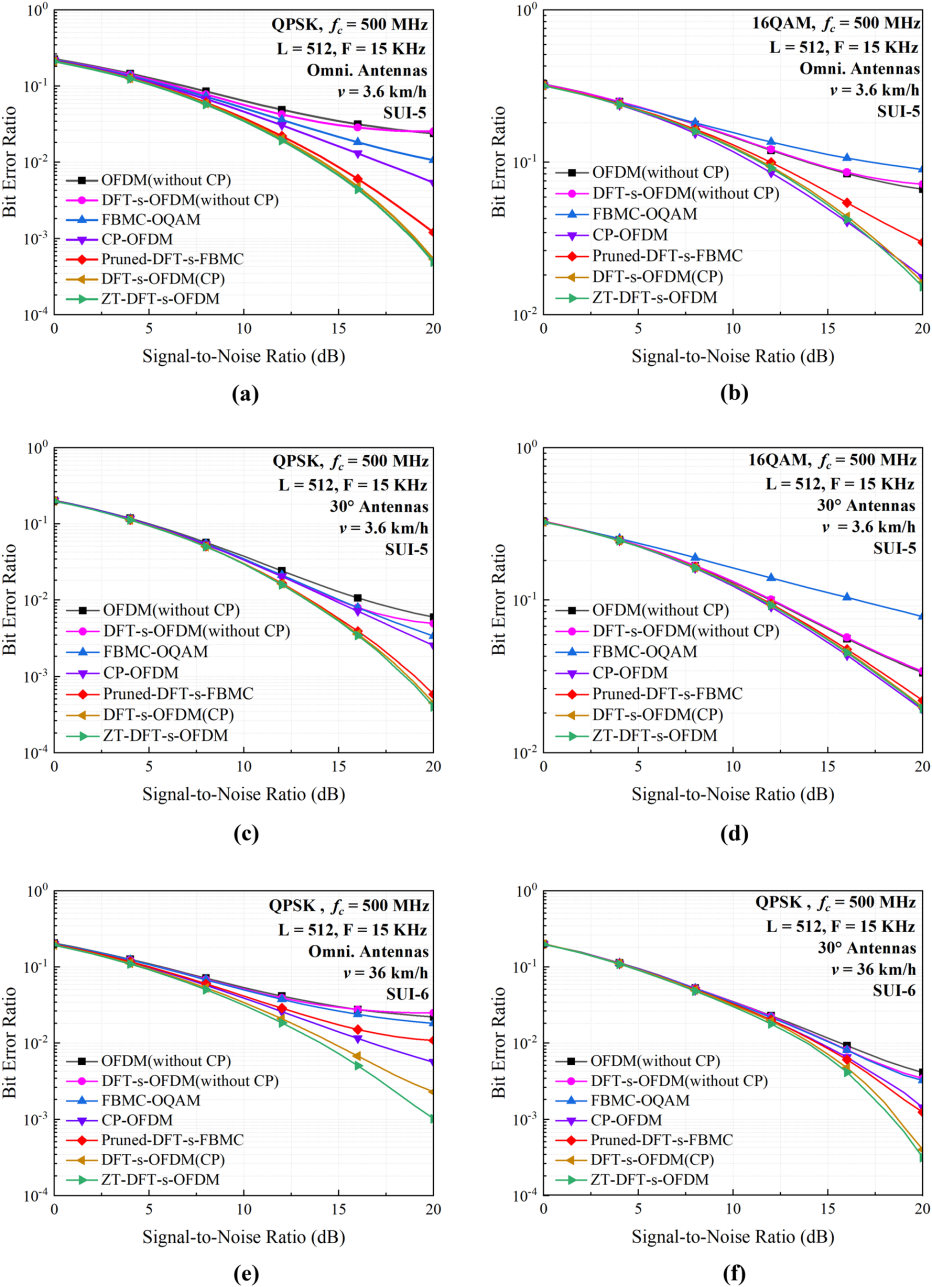
Simulated BER performance for different multi-carrier schemes under different conditions. For a fair comparison, we set the carrier frequency *f*_*c*_, the subcarrier spacing *F* and the subcarrier number *L* to constant values. (**a,b**) Are the BER performance for QPSK and 16QAM modulations under the SUI-5 channel using the omnidirectional antenna, respectively. (**c,d**) are the BER performance for QPSK and 16QAM modulations under the SUI-5 channel using the 30° directional antenna, respectively. (**e,f**) Are the BER performance for QPSK modulation under the SUI-6 channel, using the omnidirectional and directional antennas, respectively.

Figure 6 shows the simulated BER performance for various multi-carrier communication schemes, where the QPSK and 16QAM modulation methods are both analyzed. As clearly shown in Fig. 6a,b, the DFT-s-OFDM and ZT-DFT-s-OFDM schemes achieve the best BER performance due to the insertion of long protection intervals. Conventional OFDM and DFT-s-OFDM systems without CP protection have much higher BERs, and gradually achieve BER saturation with the increase of signal-to-noise ratio (SNR) at the receiver. This is because that the ISI other than the noise plays dominant role in deteriorating the BER performance for higher SNR values. In the same condition, the Pruned-DFT-s-FBMC scheme without any protection interval can achieve relatively good BER performance, only slightly worse than that of the DFT-s-OFDM and ZT-DFT-s-OFDM systems with long CP protections. This is due to the fact that a good TFL greatly enhances the ability of the signal to against ISI and ICI^19,26^ thus significantly reducing the BER. The BER analysis results are similar for the QPSK and 16QAM modulations, though the latter exhibits poorer BER performance due to the use of higher order modulation.

For the second communication solution for emergency rescue, there are two possible motion modes for the UAVs. One mode is that the UAVs move at relatively low speeds or just hover at a certain altitude, for which the SUI-5 channel model is still applicable. Being different from the first solution, however, directional antennas with relatively large volumes can be utilized for increasing the communication distance. For the low-speed mode, the movement speed of the drone UAV is still set to 3.6 km/h for a fair comparison. As depicted in Fig. 6c,d, using directional antennas with assumed half-power beam width of 30° can remarkably enhance the transmission reliability for all multi-carrier communication schemes. The simulated results show that the performance ranking is almost unchanged for different multi-carrier transmission schemes, but their BER performance are all improved substantially. This is because that the transmitter is located at a higher altitude as same as that of the UAV, which remarkably increases the probability of line-of-sight transmission. Additionally, the narrow beamwidth of 30° directional antenna helps to reduce the multipath effects thus improving the received signal quality.

Another mode of the UAV assisted solution is the relatively high-speed movement mode. Considering the Doppler shift effects caused by high motion speed for this mode, the SUI-6 channel model is utilized for analysis. For the SUI-6 channel, the multipath delay related parameters are shown in Table 1. Compared to the SUI-5 channel, the SUI-6 channel has much higher multipath time delay in addition to the Doppler effects, thus imposing bigger challenge to the robustness of the communication link. Since the maximum multipath time delay is 20 µs for the SUI-6 channel, longer protection intervals need to be added to against the influence of multipath delay. Accordingly, the lengths of the CP and ZT are set to *T*/3 = 22.2 µs, only a little longer than the maximum multipath delay. Except for different speed and channel models, other parameter sets are as same as that for the low-speed mode.

As depicted in Fig. 6e,f, the BER performance of DFT-s-OFDM and ZT-DFT-s-OFDM schemes are still better than other multi-carrier schemes, which is slightly degraded as compared with that for the low-speed mode. This mainly results from the relatively higher ICI caused by the Doppler frequency shift. On the other hand, the FBMC-OQAM and Pruned-DFT-s-FBMC schemes suffer from severe ISI due to the lack of protection interval, which exhibit relatively poor BER performance but still outperform the OFDM and DFT-s-OFDM schemes without CP. This phenomenon has indicated that, while the joint TFL of the FBMC-OQAM scheme can suppress the ISI and ICI to some extent, the long multipath delay for SUI-6 channel exceeds the tolerance of the FBMC-OQAM system thus leading to degraded BER performance. By comparing Fig. 6c,e, it is found that the ZT-DFT-s-OFDM scheme has a lower BER as compared with that of the DFT-s-OFDM scheme. This is because that the insertion of ZT and ZH allows the system to improve the TFL thus further improving the ability of against ICI. As depicted in Fig. 6f, the BER of each waveform is reduced to some extent if using a 30-degree directional antenna on a UAV with auxiliary communication. Combining the simulation results of BER for these modes, it is obvious that the use of directional antennas makes the beam more concentrated in a certain direction. This slightly reduces the effect of multipath component, which is beneficial to improving the reliability and transmission distance of the signal.

### Transmission distance analysis

Another crucial consideration for a communication link in the mountainous and dense forest environment is the transmission distance. Table 2 shows the communication system parameters commonly used in practical engineering applications.

**Table 2:** The system parameters for transmission distance simulation.

System parameter	Value	System parameter	Value
Subcarrier number	512	Low motion speed	1 m/s
Subcarrier spacing	15 kHz	High motion speed	10 m/s
Carrier frequency	500 MHz	Transmitting power *p*_*t*_	37 dBm
Transmitting antenna gain *G*_*t*_	3.5 dB (Omni.)	Transmitting antenna height *h*_*tx*_	10 m
	15 dB (30° directional)		50 m (UAVs)
Receiving antenna gain *G*_*r*_	3.5 dB (Omni.)	Receiving antenna height *h*_*rx*_	3 m
Modulation technique	QPSK	Receiver sensitivity *r*_*s*_	− 100 dBm

**Figure 7:**
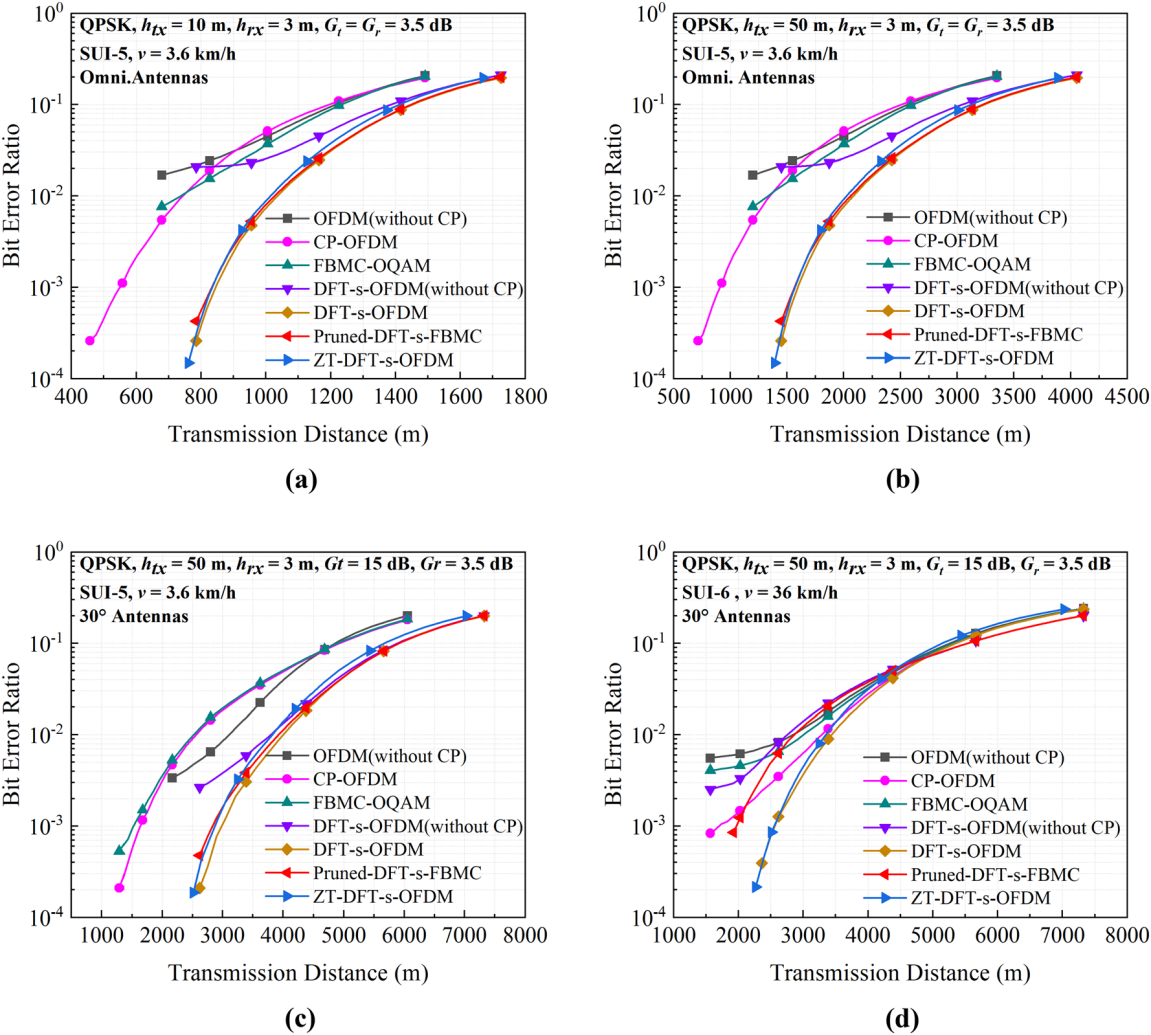
Simulated link BER performance versus the transmission distance for the cases of: (**a,b**) are the BER performance when the transmitting antenna height is 10 m and 50 m, respectively. (**c**) Shows the performance of the15 dB gain antenna. (**d**) Changes the SUI-6 sub-channel model and the speed to 36 km/h of the UAV.

Figure 7 shows the simulated BER versus the transmission distance for different multi-carrier communication schemes. It is seen that three multi-carrier schemes, i.e., the DFT-s-OFDM with CP, the ZT-DFT-s-OFDM and Pruned-DFT-s-FBMC, achieve the longest transmission distances under the same conditions. This is because that those three schemes have the lowest PAPR, which effectively increase the average transmitting power and thus received SNR. Provided the transmitting antenna height is changed from 10 to 50 m, the maximum transmission distance is approximately doubled as depicted in Fig. 7a,b. Usually, it is difficult for the rescuers to place an antenna at that height in the mountainous and dense forest environment. Even so, a very satisfactory transmission distance is still not achieved. To solve this problem, the UAV acting as a transponder with the conformal directional antenna can be raised to the desired altitude for auxiliary communication. This is one of the reasons why we propose the second solution. Figure 7c shows that if increasing the gain of the transmitting antenna to 15 dB, the transmission distance can be at least three times larger than that for the first solution. Finally, the results for the high-speed motion mode is also provided as depicted in Fig. 7d. Although the transmission performance is degraded due to the Doppler effects caused by high-speed motion, the DFT-s-OFDM and ZT-DFT-s-OFDM schemes still achieve an effective transmission distance of 2000 m. Due to the higher channel delay of SUI-6 corresponding to this motion mode, the Pruned-DFT-s-FBMC system without any added protection interval receives a serious impact and is no longer the optimal transmission waveform.

Table 3 shows the comprehensive performance comparison between different multi-carrier communication schemes in the mountainous and dense forest environment. The choice of an optimal waveform should be a combination of multiple consideration factors. It is clearly that the optimal waveform is different for different situations. For the first solution, the optimal waveform is Pruned-DFT-s-FBMC, which achieves the highest information rates of 15.36 MHz and a distance of at least 840 m. For the low-speed motion mode of the second solution, the Pruned-DFT-s-FBMC scheme achieves the same information rate and a transmission distance of at least 2950 m, which is still considered to be the optimal waveform. For the high-speed motion mode of the second solution, the ZT-DFT-s-OFDM scheme achieves the longest transmission distance of at least 2640 m, but its information rate is only around 12.29 Mbps. In comparison, the Pruned-DFT-s-FBMC scheme makes a relatively good compromise between the communication speed and distance. By performance comparison, it is shown that Pruned-DFT-s-FBMC scheme has generally the best link performance in the mountainous and dense forest environment.

**Table 3:** Performance comparison between various multi-carrier waveforms.

	Information rates (Mbps)	PAPR (dB)	Transmission distance (m) (first solution)	Transmission distance (m) (second solution)	
				Low speed	High speed
OFDM (without CP)	15.36	11.3	< 500	< 1500	< 1000
DFT-s-OFDM (without CP)	15.36	7.6	< 500	< 1500	< 1000
CP-OFDM	12.288	11.3	550	1600	1700
DFT-s-OFDM	12.288	7.6	830	3000	2550
ZT-DFT-s-OFDM	12.288	8.4	850	2850	2644
FBMC-OQAM	15.36	11.3	< 500	1500	$<$ 1000
Pruned-DFT-s-FBMC	15.36	7.6	840	2950	2000

### Performance analysis with channel coding

**Figure 8:**
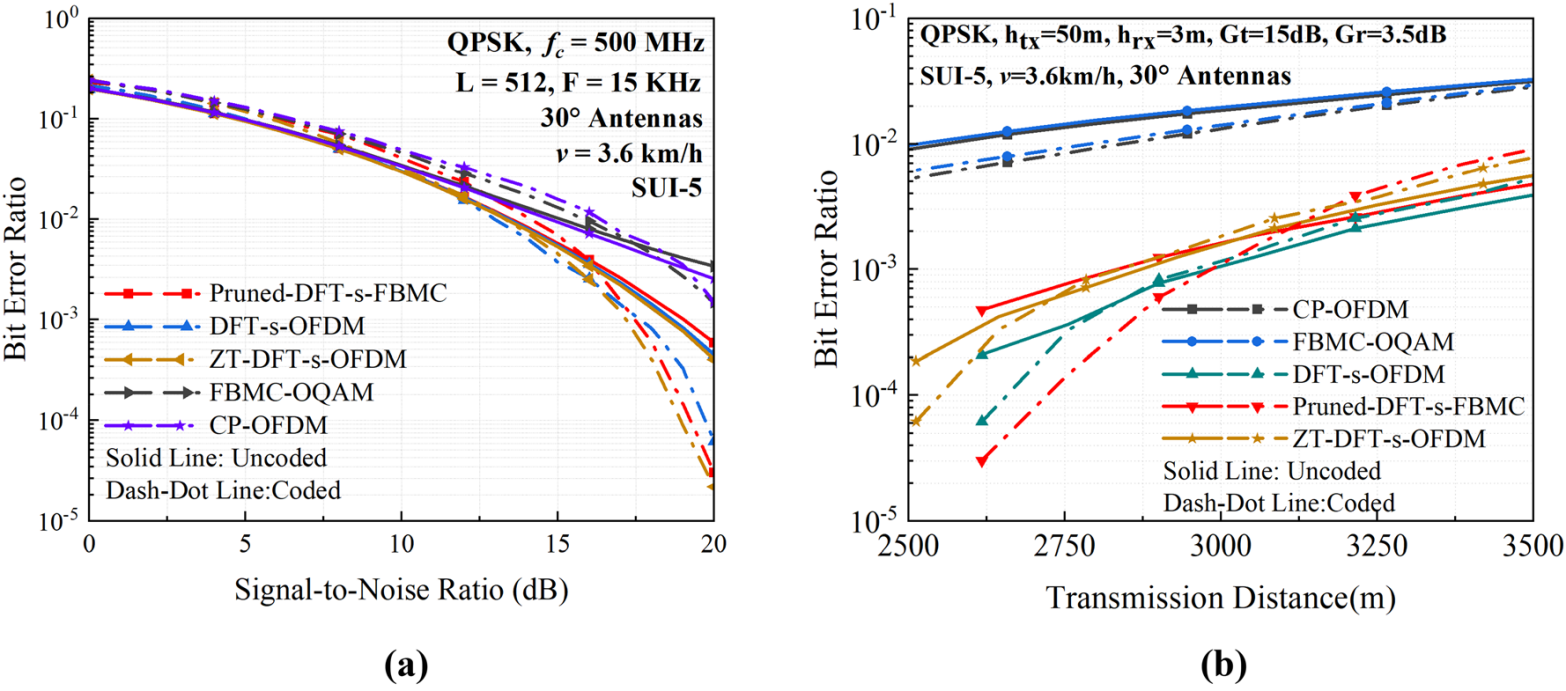
Simulated BER performance with and without LDPC coding for different multi-carrier waveforms. (**a**) BER versus SNR; (**b**) BER versus transmission distance.

Considering that practical communication systems typically employ forward error coding to improve the link quality, the BER performance after using low-density parity check (LDPC) coding has been investigated for different multi-carrier communication schemes in the mountainous and dense-forest environment. According to the Consultative Committee for Space Data Systems standard^34^, the code rate and iteration times of LDPC are set to 7/8 and 6, respectively. Since it is extremely time-consuming to accomplish computation for all cases, we have chosen some typical parameter sets for comparative simulation analysis as representative. The simulation results are shown in Fig. 8. It is clearly seen from Fig. 8a that the BER performance after LDPC coding can be improved for higher SNR values. As the coded BER drops to 10^–3^ or less, a waterfall region is observed for the DFT-s-OFDM, Pruned-DFT-s-FBMC and ZT-DFT-s-OFDM schemes. Compared to that for those three waveforms, the improvement in BER performance is much less for the FBMC-OQAM and CP-OFDM schemes even when the SNR is as high as 20 dB. This is because that when the uncoded BER is higher than a certain threshold (typically 10^–2^), LDPC coding is no longer effective for correcting the erroneous bits and even deteriorates the BER performance in some situation. Since the OFDM and DFT-s-OFDM without CP exhibits the uncoded BER as high as 10^–1^ to 10^–2^, which cannot be improved as the increase of SNR due to the saturation effect. Hence, the results for those two schemes are not shown in Fig. 8a. The simulated BER performance versus transmission distance is depicted in Fig. 8b, which clearly shows that the BER values are considerably reduced after LDPC coding for the DFT-s-OFDM, Pruned-DFT-s-FBMC and ZT-DFT-s-OFDM waveforms, while changing less for other two schemes. Moreover, the difference in coded BER performance tends to be bigger than the uncoded ones for different multi-carrier schemes at a same transmission distance.

## Conclusion

We have presented the comparative studies on various multi-carrier transmission schemes in the mountainous and dense forest environment. It is found that chasing the performance in some aspect would inevitably lead to weakening the performance in other aspects. The Pruned-DFT-s-FBMC system achieves the lowest PAPR and longest transmission distance without any additional overhead, while its BER is slightly higher in the case of ultra-high delay channels and higher order modulation. The ZT-DFT-s-OFDM scheme achieves a lower BER, but achieving lower information rate due to its long protection interval and slightly less distance as compared with that of the Pruned-DFT-s-FBMC and DFT-s-OFDM systems. Erroneous correction using LDBC coding is more effective for those three schemes as compared with other ones. In general, there is a trade-off between the reliability and validity in communication systems. All in all, we believe that the Pruned-DFT-s-FBMC scheme is more robust and efficient in the mountainous and dense forest environment, especially for the QPSK modulation mode for UAV-assisted communication.

## Data Availability

The datasets generated and analyzed during the current study are available from the corresponding author on reasonable request.
